# Ternary logic decoder using independently controlled double-gate Si-NW MOSFETs

**DOI:** 10.1038/s41598-021-92378-7

**Published:** 2021-06-21

**Authors:** Seong-Joo Han, Joon-Kyu Han, Myung-Su Kim, Gyeong-Jun Yun, Ji-Man Yu, Il-Woong Tcho, Myungsoo Seo, Geon-Beom Lee, Yang-Kyu Choi

**Affiliations:** School of Electrical Engineering, Korea Advanced Institute of Science and Technology (KAIST), 291 Daehak-ro, Yuseong-gu, Daejeon, 34141 Republic of Korea

**Keywords:** Nanoscience and technology, Nanoscale devices

## Abstract

A ternary logic decoder (TLD) is demonstrated with independently controlled double-gate (ICDG) silicon-nanowire (Si-NW) MOSFETs to confirm a feasibility of mixed radix system (MRS). The TLD is essential component for realization of the MRS. The ICDG Si-NW MOSFET resolves the limitations of the conventional multi-threshold voltage (multi-*V*_th_) schemes required for the TLD. The ICDG Si-NW MOSFETs were fabricated and characterized. Afterwards, their electrical characteristics were modeled and fitted semi-empirically with the aid of SILVACO ATLAS TCAD simulator. The circuit performance and power consumption of the TLD were analyzed using ATLAS mixed-mode TCAD simulations. The TLD showed a power-delay product of 35 aJ for a gate length (*L*_G_) of 500 nm and that of 0.16 aJ for *L*_G_ of 14 nm. Thanks to its inherent CMOS-compatibility and scalability, the TLD based on the ICDG Si-NW MOSFETs would be a promising candidate for a MRS using ternary and binary logic.

## Introduction

Scalability is one of the most important concerns in complementary metal–oxide–semiconductor (CMOS) device and circuit design^1^. Over the past several decades, the metal–oxide–semiconductor field-effect transistor (MOSFET) has been continuously scaled down to achieve higher performance and higher packing density with lower cost. An undesirable consequence of this aggressive down-scaling has been the appearance of adverse short-channel effects (SCEs), as well as increasingly challenging fabrication limits. To mitigate the SCEs, and continue further down-scaling, innovative device structures such as FinFETs, gate-all-around (GAA) FETs and a nanosheet (NS) based FET, have been introduced^2–6^. These new device structures have been able to suppress the off-state current (*I*_off_), which is fatal to the power consumption of a chip in the stand-by state. However, even with the above structural innovations, there are still limits that require device and process parameters to be continuously optimized.

Another approach to improving on-state current (*I*_on_) and chip performance has focused on the use of new materials, such as strained Si/SiGe and III–V compound semiconductors^7–9^. Although the new materials have advantages, silicon is still the most attractive material when CMOS-compatibility with low-cost is considered^10^.

In the meanwhile, multi-valued logic (MVL) has also been considered promising architecture to overcome the MOSFET scaling limitations from a circuit point of views. The MVL system can reduce the burden of circuit complexity inherent to binary-based logic circuits, by converting a multiple-output Boolean function into a single-output multiple-valued function^11,12^. In one particular case, a ternary-based logic circuit reduced the total cost and power consumption by minimizing the number of required inputs, resulting in the simplification of metal interconnection, compared to other MVL systems^13^.

In spite of these potential advantages, the practicality of ternary logic design heavily relies on the availability of the device and circuitry, which must be compatible with present-day binary CMOS technologies^12^. Binary operation has been the mainstay of modern computing system. To take full advantage of ternary logic, a mixed radix system (MRS) using both ternary and binary logic would be more suitable, rather than exclusively using ternary logic. To implement a MRS, conversion from a ternary code to a binary code is essential and vice versa^14–16^. This requires a ternary logic decoder (TLD). Logic blocks such as a ternary logic multiplexer (TLM), a TLM-based half adder and comparator can be implemented based on the TLD^17^.

To realize a TLD, a logic scheme for multi-threshold voltage (multi-*V*_th_) is necessary^18^. There have been two approaches used to implement a multi-*V*_th_ scheme. One uses physical methods, by tuning the work-function of a metal gate^19^ and by modulating the channel or body doping concentration by ion implantation^20^. The other utilizes electrical methods, applying back bias to a body in the MOSFET or potential redistribution in a gate electrode^21^. However, the aforementioned methods have several challenging issues. The gate work-function engineering increases process complexity, and limits the spectrum of potential materials, which can lead to a work function variation (WFV) problem given the variability in grain size^22^. Adjustment of *V*_th_ by ion implantation also involves issues with complementary dopants, accurate depth and concentration control, diffusivity, implantation-induced damages, and random dopant fluctuations. Moreover, channel doping concentration has little effect on *V*_th_ control for a thin gate dielectric and a fully-depleted thin-body channel^23–25^.

With the electrical approach, additional static back bias in the bulk planar MOSFET increases parasitic capacitance and leakage, which can degrade device performances. In any case, fine tuning the *V*_th_ is not an easy task, because *V*_th_ modulated by applied static back bias does not follow a linear relationship. In addition, the static back bias can degrade *I*_off_ and subthreshold swing (*SS*). For the gate electrode potential redistribution method, another challenging issue has been observed, an increase in static power consumption^21^.

In another approach, a previous study attempted to control *V*_th_ dynamically by using the four-terminals of an independently controlled double-gate (ICDG) FinFET^26^^─^^31^. Such a novel device could mitigate the abovementioned problems by allowing multi-*V*_th_. But, there have been no reports of using ICDG devices for TLD so far. A primary goal of the present work is to demonstrate a TLD composed of ICDG silicon-nanowire (Si-NW) MOSFETs, and to confirm the feasibility of manufacturing a MRS chip. Because of their inherent CMOS-compatibility and simplicity, the proposed TLD would be a promising option for realizing a mixed radix circuit.

## Results and discussion

Supplementary Fig. S1 shows a simplified sequence of the experimental details. N-channel ICDG Si-NW MOSFETs were fabricated, modeled, and fitted semi-empirically with the aid of SILVACO ATLAS TCAD simulator^32^. Thereafter, a P-channel ICDG Si-NW MOSFET was regenerated as a counter-part of the N-channel by simulations. Following device-level simulations, further analyses of the TLD circuit performance and power consumption were conducted using ATLAS mixed-mode TCAD simulations to predict the TLD behaviors at an elementary circuit level, as an extension. All of the abbreviations and nomenclature of variables are summarized in Supplementary Table S1.

**Figure 1:**
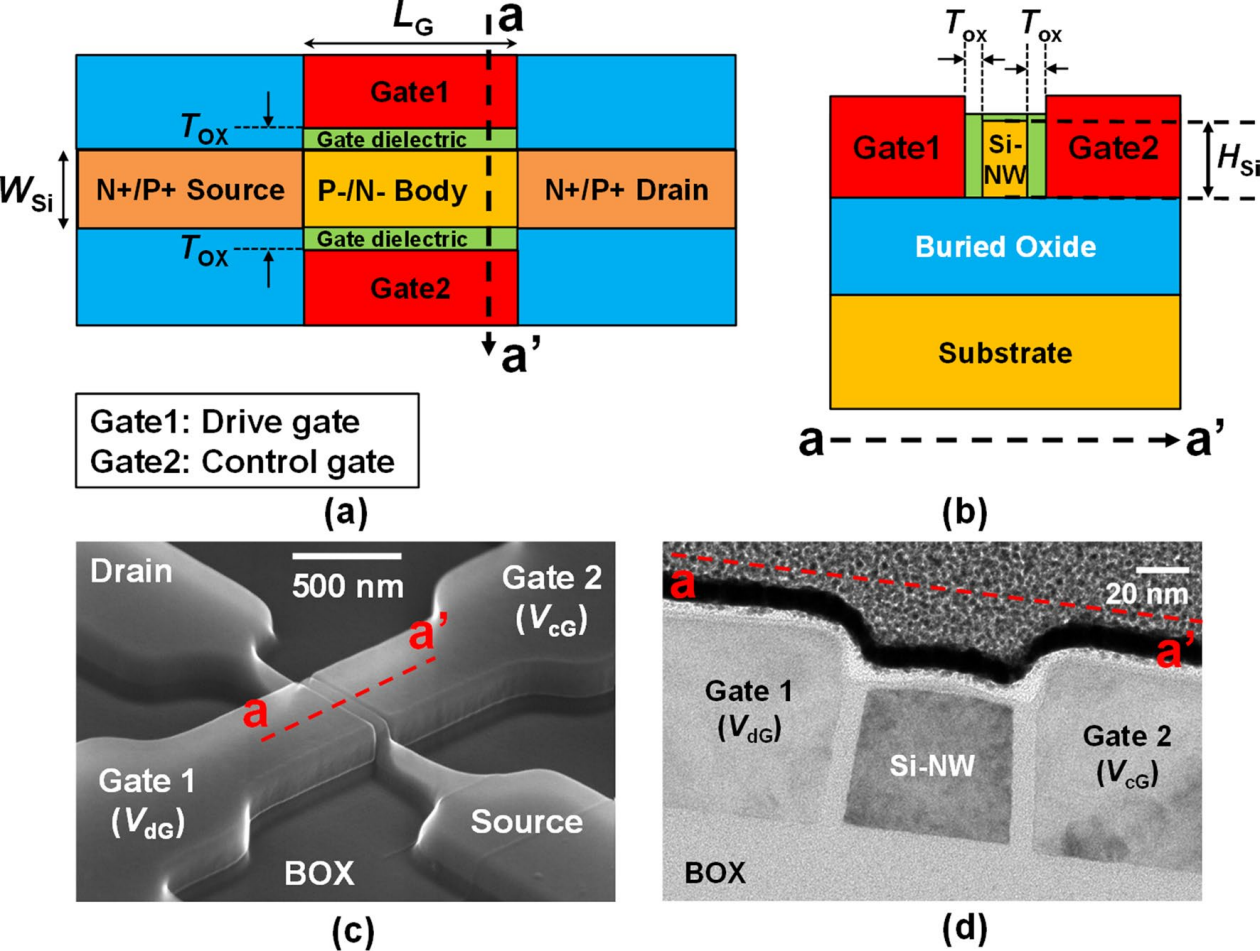
Overall structure of the ICDG Si-NW MOSFET. **(a)** Cross-sectional schematic of the ICDG Si-NW MOSFET along with the channel direction. **(b)** Cross-sectional schematic of the ICDG Si-NW MOSFET along with the gate direction. **(c)** SEM image of the fabricated ICDG Si-NW MOSFET. **(d)** Cross-sectional TEM image of the fabricated ICDG Si-NW MOSFET along with the gate direction.

Fabrication process of the ICDG Si-NW MOSFET is summarized in Supplementary Fig. S2. Figure 1 shows a schematic of the ICDG Si-NW MOSFET and scanning electron microscopy (SEM) and transmission electron microscopy (TEM), images of the fabricated ICDG Si-NW MOSFET. The fabricated ICDG Si-NW MOSFET was composed of two gates, a drive gate and a control gate, positioned at each sidewall of the Si-NW to control the current flowing in the Si-NW. The drive gate turns the channel on or off. The control gate modulates threshold voltage as a body electrode does in a conventional bulk-MOSFET. The fabricated device had a Si-NW width (*W*_Si_) of 70 nm, a gate length (*L*_G_) of 500 nm, a Si-NW height (*H*_Si_) of 50 nm and a gate dielectric thickness (*T*_ox_) of 10 nm. The dimensions of the modeled device were the same as the fabricated device. Source, body, and drain doping concentration were set to 1 $\times$ 10^20^ cm^–3^, 1 $\times$ 10^15^ cm^–3^, 1 $\times$ 10^20^ cm^–3^, respectively. Various models such as Schockly-Read-Hall (SRH), bandgap narrowing (BGN), Fermi–Dirac (FERMI), energy balance model (EBM), non-local band-to-band tunneling (BTBT), trap-assisted tunneling (TAT), and quantum effect (QUANTUM for electrons and P.QUANTUM for holes) were utilized for the simulations. The P-channel ICDG Si-NW MOSFET was modeled in the same manner as the N-channel device, except for the doping polarity and *H*_Si_. The dopant of the P-channel device was the opposite that of the N-channel device, and the *H*_Si_ was doubled considering the difference in carrier mobility of electrons and holes.

**Figure 2:**
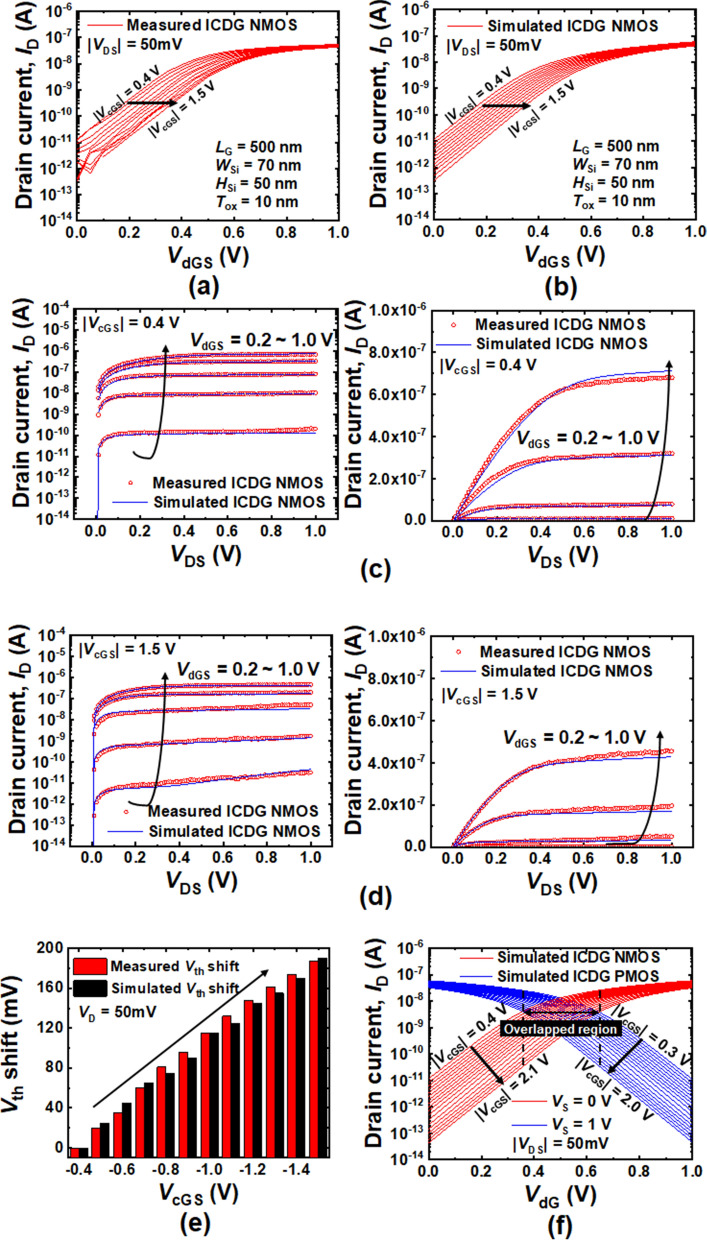
**(a)** Measured *I*_D_-*V*_dGS_ characteristics from the fabricated ICDG NMOS for various **|***V*_cGS_**|**. **(b)** Semi-empirically simulated *I*_D_-*V*_dGS_ characteristics according to various **|***V*_cGS_**|**. Superimposition of measured and simulated *I*_D_-*V*_DS_ characteristics of N-channel for **(c) |***V*_cGS_**|**= 0.4 V and **(d)** 1.5 V on the log-scaled *y*-axis and linear-scaled *y*-axis. **(e)** Linear *V*_th_ shift by *V*_cGS_. **(f)** Simulated *I*_D_-*V*_dGS_ characteristics of both ICDG NMOS and PMOS for various **|***V*_cGS_**|**.

Figure 2(a) and (b) show the *I*_D_-*V*_dGS_ characteristics of the measured and simulated N-channel ICDG Si-NW MOSFET, respectively. The *I*_D_–*V*_dGS_ curves are shown for various **|***V*_cGS_**|**. Measured and simulated *I*_D_–*V*_DS_ characteristics of N-channel ICDG Si-NW MOSFET are superimposed for *V*_cGS_ =  0.4 V (Fig. 2c) and *V*_cGS_ = − 1.5 V (Fig. 2d) on the log-scaled *y*-axis and linear-scaled *y*-axis. They (measured and simulated) are very similar to each other. Because individual gate addressing is possible thanks to the use of two local gates, the channel potential of the Si-NW can be controlled independently^33^. One of the two gates is used to sweep the gate voltage of the drive gate while a constant voltage is applied to the other gate, which is the control gate to precisely tune the channel potential of the Si-NW. A shift in *V*_th_ and its multiple values by *V*_cGS_ are shown in Fig. 2e. The *V*_th_ shift by *V*_cGS_ has linear relationship. The results are consistent with the previously reported data^26–31^. As the **|***V*_cGS_**|** increased, the *I*_D_-*V*_dGS_ curve shifted rightward in parallel and the *V*_th_ was increased. Herein, leakage current is defined as off-state current at *V*_dGS_ of 0 V. The measured leakage current from the fabricated ICDG Si-NW NMOSFET was decreased from 10 pA to 300 fA as the |*V*_cGS_| was increased from 0.4 V to 1.5 V. Figure 2f shows the symmetrically overlaid *I*_D_-*V*_dGS_ curves of the simulated N-channel and P-channel ICDG Si-NW MOSFET. Here, there is a wide overlapped region between the N-channel device and the P-channel device. This wide overlapped region stably creates a intermidiate output signal (output ‘1’), which enables the TLD. It becomes wider as the applied **|***V*_cGS_**|** increases.

**Figure 3:**
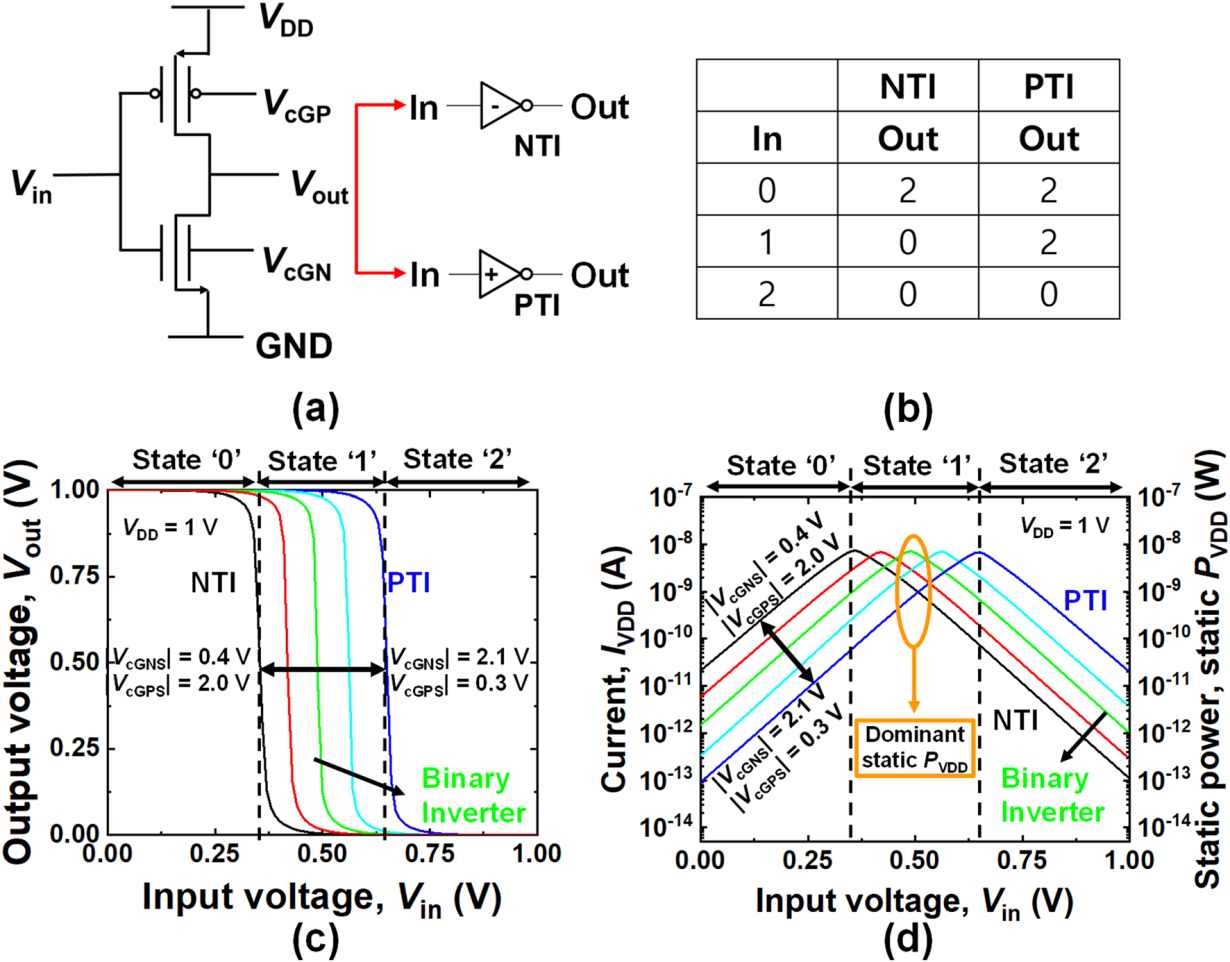
**(a)** Complementary inverter circuits composed of ICDG NMOS and PMOS with each control gate. They can serve as the NTI and the PTI by adjusting *V*_cGN_ and *V*_cGP_. **(b)** Truth table of the NTI and PTI. **(c)** Input–output voltage transfer curves (VTCs) of the inverter circuit for various **|***V*_cGNS_**|** and **|***V*_cGPS_**|**. **(d)** Current (*I*_VDD_) from the *V*_DD_ node to the ground node versus input voltage (*V*_in_) and power consumption (*P*_VDD_) versus input voltage (*V*_in_).

Note that there are negative ternary inverters (NTI) and positive ternary inverters (PTI) in ternary logic. Its output state becomes ‘0’ or ‘2’ when the input state is ‘1′^18^ as shown in Fig. 3b. The proposed TLD was composed of NTI, PTI, and negative ternary NOR (NTNOR)^18^. To implement the TLD, the characteristics of NTI and PTI should be confirmed.

As shown in Fig. 3a, the ternary inverter circuitry is similar to a typical binary inverter except for the use of the control gates. The complementary circuit shown in Fig. 3a can become both the NTI and the PTI by adjusting *V*_cGN_ and *V*_cGP_. As shown in Fig. 3c, the voltage transfer curve (VTC) is shifted in parallel as **|***V*_cGNS_**|** increases from 0.4 V to 2.1 V and |*V*_cGPS_| decreases from 2.0 V to 0.3 V. For the NTI, an input voltage (*V*_in_) of 0.5 V (state ‘1’) is transformed to a *V*_out_ of 0 V (state ‘0’) because |*V*_th_| in the P-channel device is higher than that of the N-channel device. In contrast, the *V*_in_ of 0.5 V (state ‘1’) is converted to a *V*_out_ of 1 V (state ‘2’) in the case of the PTI, due to the higher |*V*_th_| of the N-channel device.

Figure 3d shows the *I*_VDD_ and *P*_VDD_ characteristics versus the *V*_in_. The *I*_VDD_ increased and decreased exponentially as the N-channel and P-channel devices started to turn on and turn off, respectively, at a certain voltage. Peak points of the *I*_VDD_ curve at a certain voltage, were shifted as the **|***V*_cGNS_| and |*V*_cGPS_| changed. *P*_VDD_ can be obtained by multiplying the *I*_VDD_ by *V*_DD_ for various *V*_in_, as shown in the 2nd *y*-axis in Fig. 3d. We can average *P*_VDD_ for three states with a different weighting factor: *w*_0_ for state ‘0’, *w*_1_ for state ‘1’ and *w*_2_ for state ‘2’. This is represented by < *P*_VDD_ > _avg_ = (*w*_0_⋅*P*_VDD_|_state=0_ + *w*_1_⋅*P*_VDD_|_state=1_ + *w*_2_⋅*P*_VDD_|_state=2_)/(*w*_0_ + *w*_1_ + *w*_2_). This averaged *P*_VDD_ is approximated to (1/3)⋅*w*_1_⋅*P*_VDD_|_state=1_ under the condition of *w*_0_ = *w*_1_ = *w*_2_, because the *P*_VDD_|_state=2_ and *P*_VDD_|_state=0_ are much smaller than the *P*_VDD_|_state=1_. It should be noted that *I*_VDD_ for state ‘1’ is much larger than that at the state ‘0’ and ‘2’. When the curve (green line) of a typical binary inverter in Fig. 3d is shifted to the NTI or the PTI, the *P*_VDD_|_state=1_ decreases. Accordingly, the total *P*_VDD_ decreases.

**Figure 4:**
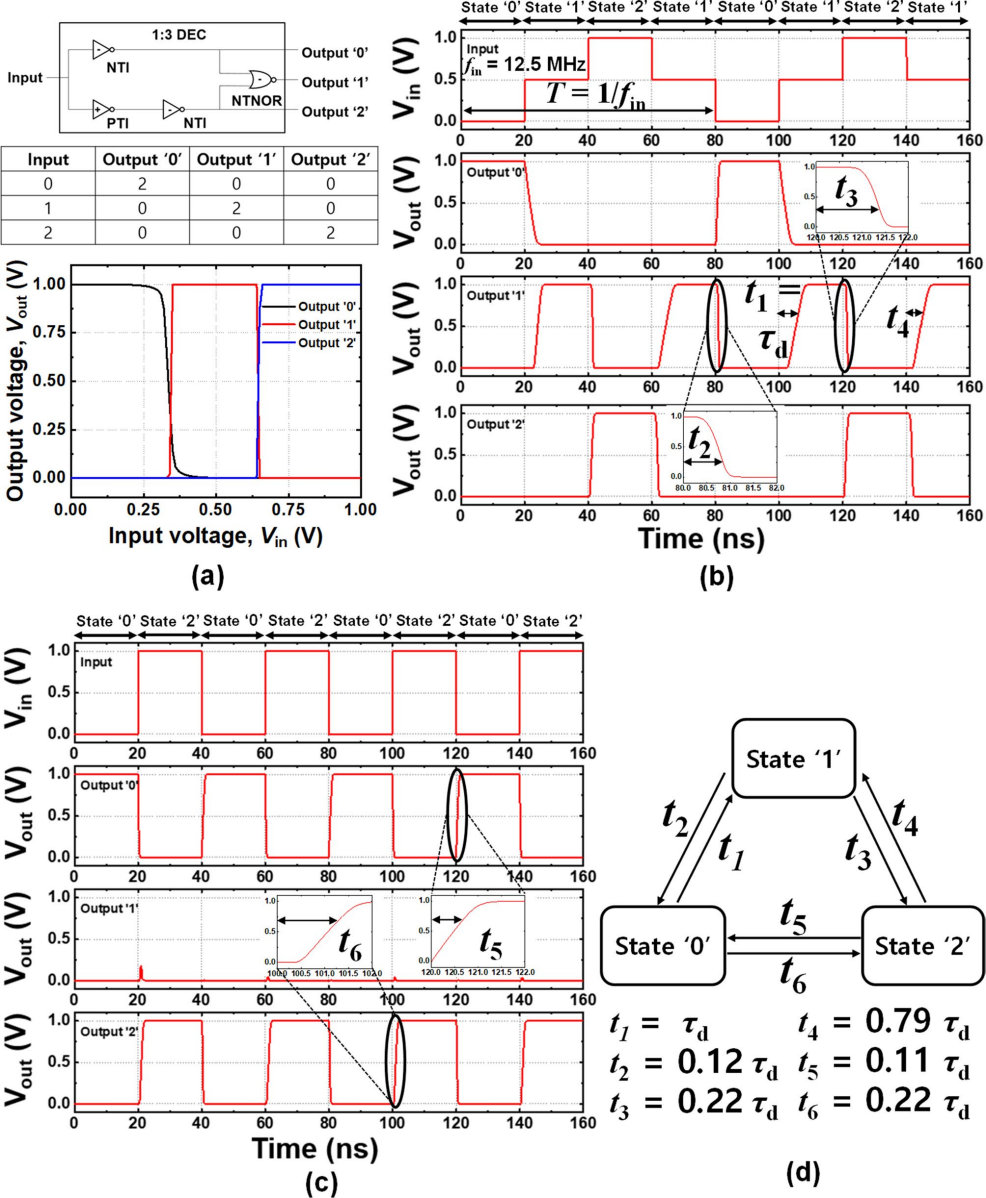
**(a)** 1-to-3 ternary logic decoder circuit composed of NTI, PTI and NTNOR and the VTCs of output ‘0’, output ‘1’ and output ‘2’. **(b)** Transient ternary responses of output ‘0’, output ‘1’, output ‘2’ versus time (*f*_in_ = 12.5 MHz). **(c)** Transient binary responses for direct alteration between states ‘2’ and ‘0’. **(d)** Diagram of state transition with propagation delay time.

A 1-to-3 TLD circuit was designed to examine the feasibility of the ICDG Si-NW MOSFET for the TLD. The TLD consisted of 10 complementary ICDG Si-NW MOSFETs. Figure 4a shows the TLD logic circuit and its VTCs for an output ‘0’, output ‘1’, and output ‘2’ versus the *V*_in_. The NTNOR CMOS circuitry was the same as the typical binary NOR except for the use of the control gates. The device models for the NTNOR were identical to the device models described in Fig. 2. As shown in the VTC graph in Fig. 4a, the TLD stably accepts voltage near 0.5 V as an input then it produces the third output voltage (output ‘1’). The behaviors of the TLD were verified using the ATLAS mixed-mode TCAD simulations, as shown in Fig. 4b and c. Voltage levels of 1 V (*V*_DD_), 0.5 V (half *V*_DD_), and 0 V (*V*_SS_) are equivalent to a logic value of ‘2’, ‘1’, and ‘0’, respectively. Figure 4d shows a diagram of the state transition with propagation delay time. Note that the transition time is related to on-current of a MOSFET. According to the result of Fig. 4d, *t*_2_ (‘1’ → ‘0’) was almost the same as *t*_5_ (‘2’ → ‘0’) because both the *t*_2_ and *t*_5_ depend on the on-current of an n-channel pull-down transistor (*I*_on, ‘0’_). Likewise, *t*_3_ (‘1’ → ‘2’) was the same as *t*_6_ (‘0’ → ‘2’) because both the *t*_3_ and *t*_6_ depend on the on-current of a p-channel pull-up transistor (*I*_on, ‘2’_). *t*_1_ and *t*_4_ are dominated by on-current (*I*_on, ‘1’_) at an intermediate state. *I*_on, ‘1’_ is defined as the drain current at *V*_dGS_ = *V*_DD_/2. As shown in Fig. 2f, the *I*_on, ‘1’_ was smaller than the *I*_on, ‘0’_ and *I*_on, ‘2’_. Therefore, *t*_1_ and *t*_4_ were longer than the other transition times (*t*_2_, *t*_3_, *t*_5_, and *t*_6_). Additionally, the difference between *t*_1_ and *t*_4_ was arisen from a slight disparity of *I*_D_ between *I*_on, ‘1’_ of a p-channel MOSFET and an n-channel MOSFET. The delay time (*τ*_d_) is dominated by the longest transition time (*t*_1_). To reduce the delay time, *I*_on, ‘1’_ modulated by |*V*_cGS_| should be maximized as large as possible.

**Figure 5:**
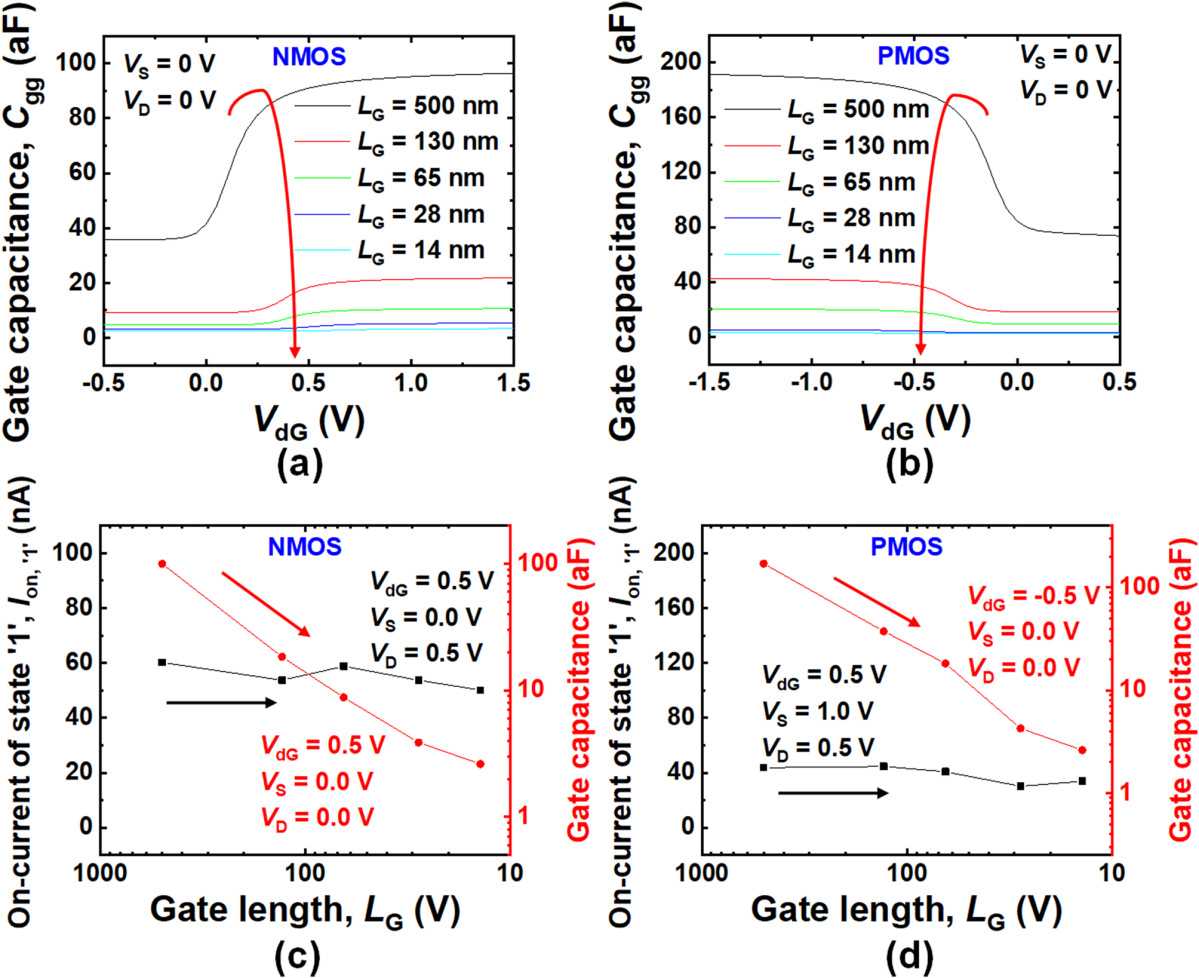
The *C*_gg_-*V*_dG_ characteristics of ICDG **(a)** NMOS and **(b)** PMOS depending on gate length (*L*_G_) reduction. *I*_on, ‘1’_ and *C*_gg_ of ICDG **(c)** NMOS and (d) PMOS according to gate length reduction.

Based on SILVACO ATLAS TCAD simulator, the scaling analysis was implemented by reducing device dimensions (*L*_G_, *W*_Si_, *H*_Si_, and *T*_ox_). The detailed information of those is shown in Supplementary Table S2. Figure 5 shows a quantitative analysis of gate capacitance (*C*_gg_) and *I*_on, ‘1’_. Figures 5a and b show the *C*_gg_-*V*_dG_ characteristics depending on *L*_G_ under certain *V*_cG_ conditions which were optimized for *V*_th_-modulation. Optimization of *V*_th_-modulation according to *V*_cG_ can be performed with consideration of two conflicting demands: maximization of speed and minimization of static power consumption. Increment of *I*_on, ‘1’_ through the *V*_cG_ modulation can result in boosting speed of the ternary logic decoder (TLD). But, excessive increment of *I*_on, ‘1’_ can adversely increase the static power consumption (*P*) of the TLD owing to a parallel shift of *V*_th_, which provokes increment of leakage current. Thus, the optimization of *V*_cG_ can be done with a well-known figure of merit, power-delay product (*PDP*) owing to the abovementioned trade-off relationship. The magnitude of *C*_gg_ for N-channel and P-channel ICDG Si-NW MOSFETs was decreased as *L*_G_ was decreased. As shown in Fig. 4c and d, as *L*_G_ was decreased, *C*_gg_ was continuously decreased, while *I*_on, ‘1’_ was maintained by *V*_th_-modulation. It means that delay time (*τ*_d_) of the TLD circuit is decreased by down-scaling.

**Figure 6:**
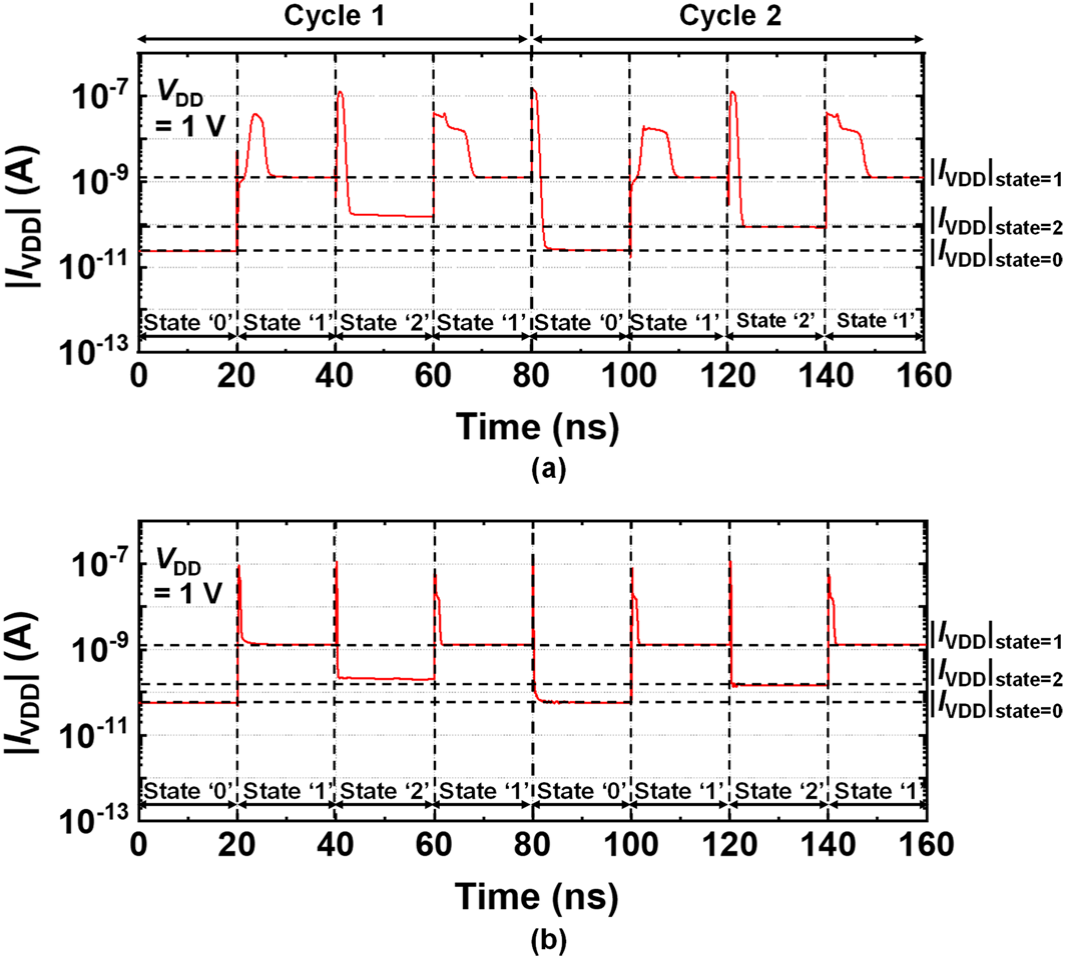
Transient response of *I*_VDD_ versus time for **(a)** *L*_G_ = 500 nm and **(b)** *L*_G_ = 14 nm.

Figure 6a and b show two transient current responses for an *L*_G_ of 500 nm and an *L*_G_ of 14 nm, respectively at input voltage frequency (*f*_in_) of 12.5 MHz. The *f*_in_ of 12.5 MHz was used from *T* = 1/*f*_in_ = 80 ns as shown in Fig. 4b first graph. When the input voltage signal abruptly switched from one state to another, charges were transferred from the power supply to the gate capacitors or load capacitors. And |*I*_VDD_| rapidly increased and temporarily overshot, thereafter it began to stabilize. As shown in Fig. 6b, the pulse width needed to induce the overshoot was significantly reduced by down-scaling from *L*_G_ = 500 nm to *L*_G_ = 14 nm. The stabilized |*I*_VDD_| depended on the applied *V*_in_ (state of input). The magnitude of the stabilized |*I*_VDD_| for the input voltage of 0.5 V (state ‘1’) was much higher than that for the other states, like the |*I*_VDD_| of the NTI and PTI shown in Fig. 3d. For an *L*_G_ = 500 nm, a total *P*_VDD_ of 6.37 nW was calculated by integrating *V*_DD_ $\times$ |*I*_VDD_(*t*)| with respect to the 2 cycle time (2* T* = 160 ns) and dividing it by the same time, i.e., $\left( {{\text{total}}\,{P_{{\text{VDD}}}}} \right)\, = \,[{V_{{\text{DD}}}} \cdot \int_{0\,{\text{ns}}}^{160\,{\text{ns}}} {\left| {{I_{{\text{VDD}}}}(t)} \right|} dt]/(160\,{\text{ns}})$.

The TLD was evaluated in terms of *τ*_d_, *P*, and *PDP*. These values were semi-empirically extracted from the simulations, which were based on the fabricated device. Theoretically, *τ*_d_ can be reduced by *V*_th_ engineering by biasing the control gate, as well as by device down-scaling. The above three metrics were changed by reducing the gate length (*L*_G_), as summarized in Table 1. As shown in Fig. 4d, the propagation delay times, *t*_1_ from state ‘0’ to state ‘1’ and *t*_4_ from state ‘2’ to state ‘1’ were longer than the other transition times (*t*_2_, *t*_3_, *t*_5_, and *t*_6_) because *I*_on, ‘1’_ is smaller than *I*_on, ‘0’_ and *I*_on, ‘2’_. In this work, the *τ*_d_ of TLD is predominantly governed by the longest transition time, *t*_1_ (*t*_1_ > *t*_4_).

**Table 1:** Power and delay time according to gate length scaling.

Gate length (*L*_G_) [nm]	Delay (*τ*_d_) [ns]	Possible operating frequency [GHz]	Power (*P*) [nW] (at *f*_in_ [MHz])	*PDP* [aJ]
500	5.57	0.045	6.37 (12.5)	35.48
130	0.95	0.263	2.68 (48.1)	2.55
65	0.44	0.568	2.20 (96.2)	0.97
28	0.18	1.389	2.14 (223.2)	0.39
14	0.085	2.941	1.90 (446.4)	0.1615

The possible operating frequency was calculated from *f* = 1 / (4*τ*_d_)^34^. Following a constant field scaling scenario with a scaling factor of *K* (> 1), *f*_in_ is proportional to *K* for the down-scaling^1^. Therefore, the *f*_in_ for a short *L*_G_ becomes *f*_in_⋅long *L*_G_/short *L*_G_. For example, an *f*_in_ of 12.5 MHz for an *L*_G_ of 500 nm can be increased to 48.1 MHz with an *L*_G_ of 130 nm. *τ*_d_ was directly extracted and *P* was extracted for each *f*_in_ predicted by the abovementioned scaling rule for an *L*_G_ of 500 nm, 130 nm, 65 nm, 28 nm, and 14 nm, as shown in Table 1. The *τ*_d_ of 5.57 ns for *L*_G_ of 500 nm was drastically reduced to 0.085 ns for an *L*_G_ of 14 nm. And the *P* of 6.37 nW for an *L*_G_ of 500 nm was also reduced to that of 1.90 nW for *L*_G_ of 14 nm. In addition, the *PDP* of 0.16 aJ was extracted from *P* of 1.9 nW and *τ*_d_ of 0.085 ns for *L*_G_ of 14 nm. The performance metrics of the proposed TLD are compared with the existing implementations, as shown in benchmarking Table 2^18,35–39^.

**Table 2:** Benchmarking table of ternary logic decoder.

	Type of devices	*V*_th_-modulation	Number of transistors	Power (*P*) [nW]	Delay (*τ*_d_) [ps]	*PDP* [aJ]
^18^	MOSFETs	Body Ion Implantation	10	0.444	> 23,000	10.2
^35^	CNTFETs	Diameter Engineering	16	11,800	8.24	97.2
^36^	MOSFETs	CMOS Double Pass Logic	12	13,000	140	1820
^37^	CNTFETs	Diameter Engineering	11	10,400	7.06	73.4
^38^	CNTFETs	Diameter Engineering	10	250,000	7.18	1795
^39^	CNTFETs	Diameter Engineering	9	9100	4.18	38.0
This work	ICDG Si-NW MOSFETs	Electrical Control Bias	10	1.90	85	0.16

The *SS* of the ICDG Si-NW MOSFET in this experiment was approximately 120 mV/dec, due to the poor interface quality of the TEOS used as the gate oxide. This can be decreased to sub-80 nm/dec replacing the thermally grown oxide or using a high-*k* dielectric material. Further improvement in the *SS* will additionally reduce *τ*_d_ and *P*.

In this study, a ternary logic decoder (TLD) to allow the conversion from a ternary code to a binary code and vice versa has been demonstrated with independently controlled double-gate (ICDG) silicon-nanowire (Si-NW) MOSFETs. Feasibility of the TLD was explored by use of semi-empirical circuit-level simulations based on the measured device-level ICDG Si-NW MOSFET characteristics. Because the ICDG Si-NW MOSFET is not only suitable for a multi-*V*_th_ scheme but also CMOS-compatible for mass-production, the proposed TLD would be a promising candidate to realize a MRS. Direct demonstration of the TLD with fully fabricated circuits is left as a further work.

## Methods

### Electrical measurements

A semiconductor parameter analyzer (B1500A) was used to characterize the fabricated ICDG Si-NW MOSFETs. The transfer characteristics of the fabricated N-channel ICDG Si-NW MOSFET were measured at a constant *V*_DS_ of 50 mV, whereas the *V*_dGS_ was swept from 0 to 1 V.

### Device modeling

The ICDG Si-NW MOSFETs were modeled and fitted with the SILVACO ATLAS TCAD simulator. A various physical models such as Schockly-Read-Hall (SRH), Bandgap Narrowing (BGN), Fermi–Dirac (FERMI), non-local Band-to-Band Tunneling (BTBT) and Trap-Assisted Tunneling (TAT) were involved.

### Circuit simulation for ternary logic decoder

Using the experimentally modeled devices, the operation, performance, and power consumption of the TLD were verified using the ATLAS mixed-mode TCAD simulations. To simulate the transient response of the TLD, *V*_DD_ and *V*_SS_ were set to 1 V and 0 V respectively.

## Supplementary Information

Supplementary Information.
