# Author Correction: Ternary logic decoder using independently controlled double-gate Si-NW MOSFETs

**DOI:** 10.1038/s41598-021-96555-6

**Published:** 2021-08-19

**Authors:** Seong‑Joo Han, Joon‑Kyu Han, Myung‑Su Kim, Gyeong‑Jun Yun, Ji‑Man Yu, Il‑Woong Tcho, Myungsoo Seo, Geon‑Beom Lee, Yang‑Kyu Choi

**Affiliations:** School of Electrical Engineering, Korea Advanced Institute of Science and Technology (KAIST), 291 Daehak‑ro, Yuseong‑gu, Daejeon, 34141 Republic of Korea

Correction to: *Scientific Reports* 10.1038/s41598-021-92378-7, published online 21 June 2021

The original version of this Article contained an error in the spelling of the author Joon-Kyu Han which was incorrectly given as Joon-kyu Han. The original Article and accompanying Supplementary Information file have been corrected.

