# Supplementary material for: Ternary logic decoder using independently controlled double-gate Si-NW MOSFETs
Source: Sci Rep. 2021 Jun 21;11:13018. doi: 10.1038/s41598-021-92378-7 (PMC8217211; doi:10.1038/s41598-021-92378-7)
Supplement: Supplementary file 1 — Supplementary Information. [file 41598_2021_92378_MOESM1_ESM.docx]

**Supplementary Information**

**Ternary Logic Decoder Using Independently Controlled Double-gate Si-NW MOSFETs**

*Seong-Joo Han^1^, Joon-Kyu Han^1^, Myung-Su Kim^1^, Gyeong-Jun Yun^1^, Ji-Man Yu^1^, Il-Woong Tcho^1^, Myungsoo Seo^1^, Geon-Beom Lee^1^, and Yang-Kyu Choi^1,a)^*

^1^School of Electrical Engineering, Korea Advanced Institute of Science and Technology, (KAIST) 291 Daehak-ro, Yuseong-gu, Daejeon 34141, Republic of Korea

1. Authors to whom correspondence should be addressed.

Email addresses: [ykchoi@ee.kaist.ac.kr](mailto:ykchoi@ee.kaist.ac.kr)

**Sequence of the experimental details.**

**Figure S1.** Sequence of mixed-mode simulations considering the measured characteristics of the fabricated N-channel ICDG MOSFET.

Fig. S1 shows a simplified sequence of the technical details of modelling for numerical simulations. N-channel ICDG Si-NW MOSFETs were fabricated, modelled, and fitted semi-empirically with the aid of SILVACO ATLAS TCAD simulator. Thereafter, a P-channel ICDG Si-NW MOSFET was regenerated as a counter-part of the N-channel by simulations. After the abovementioned device-level simulations, further analyses for the TLD circuit performance and its power consumption were conducted using ATLAS mixed-mode TCAD simulations to predict the TLD behaviors at a circuit-level, as an extension of work.

***A. Fabrication of long-channel ICDG Si-NW NMOSFETs***

Refer to Fig. S2.

***B. Electrical measurements of long-channel ICDG Si-NW NMOSFETs***

A semiconductor parameter analyzer (B1500A) was used to characterize the fabricated ICDG Si-NW MOSFETs. The transfer characteristics of the fabricated N-channel ICDG Si-NW MOSFET were measured at a constant *V*_DS_ of 50 mV, whereas the *V*_dGS_ was swept from 0 V to 1 V.

***C. Dimensional fitting to long-channel ICDG Si-NW NMOSFETs for simulation***

Measured characteristics of long-channel ICDG Si-NW NMOSFETs were modelled and fitted with the SILVACO ATLAS TCAD simulator. The dimensions of the modelled device were the same as those of the fabricated device. The fabricated device had a Si-NW width (*W*_Si_) of 70 nm, a gate length (*L*_G_) of 500 nm, a Si-NW height (*H*_Si_) of 50 nm and a gate dielectric thickness (*T*_ox_) of 10 nm. Source, body, and drain doping concentration were set to 1 $\times$ 10^20^ cm^–3^, 1 $\times$ 10^15^ cm^–3^, 1 $\times$ 10^20^ cm^–3^, respectively. Various models such as Schockly-Read-Hall (SRH), bandgap narrowing (BGN), Fermi-Dirac (FERMI), energy balance model (EBM), non-local band-to-band tunneling (BTBT), trap-assisted tunneling (TAT), and quantum effect (QUANTUM for electrons and P.QUANTUM for holes) were reflected for the simulations.

***D. Fine tuning to short-channel ICDG Si-NW NMOSFETs by simulation***

In addition to the dimensional fitting, fine tuning was applied to a short-channel as well as a long-channel ICDG Si-NW NMOSFET with consideration of other variables such as concentration-dependent channel mobility, contact resistance, source/drain extension length (*L*_SD_), etc. And their transfer characteristics of *I*_D_-*V*_dGS_ for various *V*_cGS_ and output characteristics of *I*_D_-*V*_DS_ for various *V*_cGS_ and *V*_dGS_ were regenerated for reasonably fair comparison of other previous works with the aid of SILVACO ATLAS TCAD simulator. The results of fine tuning are shown in Fig. 2(a), (b), (c), and (d) in Results and Discussion section.

***E. Calibration for ICDG Si-NW PMOSFETs by simulation via bisymmetric conversion of NMOSFETs***

As a counter-part of ICDG Si-NW NMOSFETs, electrical characteristics of the P-channel ICDG Si-NW MOSFETs were bisymmetrically produced with two considerations. One is to reverse doping polarity of gate, source, drain, and channel. The other is to reflect carrier mobility and a channel height (*H*_Si_), which is corresponding to a half of a channel width. In other words, the *H*_Si_ of a PMOSFET was doubled compared with that of an NMOSFET by considering a difference of carrier mobility between electrons and holes.

***F. Mixed-mode simulations of ICDG Si-NW NMOSFETs and PMOSFETs***

Being based on semi-empirically simulated NMOSFETs and PMOSFETs, a 1-to-3 TLD circuit was designed to explore the feasibility of the TLD. The TLD consisted of 10 complementary ICDG Si-NW MOSFETs. The operation, performance, and power consumption of the TLD were verified using the ATLAS mixed-mode TCAD simulations. To simulate the transient response of the TLD, *V*_DD_ and *V*_SS_ were set to 1 V and 0 V, respectively.

**Table S1.** Summary of abbreviations and nomenclature of variables.

**Fabrication process flow of the ICDG Si-NW MOSFET**

**Figure S2. (a)** The starting material was a commercial 8-inch p-type silicon-on-insulator (SOI) wafer. Its initial resistivity was 9 to 18 Ω·cm, with a top silicon thickness of 50 nm, and a buried oxide thickness of 140 nm. **(b)** A silicon nitride (Si_3_N_4_) layer was deposited using the low-pressure chemical vapor deposition (LPCVD) method. **(c)** To create Si-NWs, Si_3_N_4_ and Si layers were patterned by optical lithography (KrF, wavelength of 193nm) and conventional plasma etching. The width of the Si-NW was 70nm. **(d)** A tetraethylorthosilicate (TEOS) oxide of 10 nm and an n^+^ *in-situ* doped poly-crystalline silicon (poly-Si) layer of 80 nm were sequentially deposited, which served as the gate dielectric and electrode, respectively. **(e)** After the poly-Si deposition, the protruded poly-Si on the Si-NW channel was polished by a chemical mechanical polishing (CMP) process until the buried Si_3_N_4_ revealed. The Si_3_N_4_ served as an etching stopper to prevent the Si-NW from being etched during the CMP process. After patterning the gate region, the source and drain doping process was carried out by ion implantation (arsenic, energy of 30 keV and dose of 5 $\times$ 10^15^ cm^–2^). Afterwards, rapid thermal annealing (1000 ℃, 5 sec) was employed to activate the dopants. **(d)** The remaining Si_3_N_4_ on the Si-NW channel was completely removed by hot phosphoric acid. To cure damage produced by the Si_3_N_4_ wet-etching, additional oxidation was carried out at 700 ℃ for 30min in an oxygen ambient so that a thermal oxide (SiO_2_) grew up, with a thickness of 3nm.

**Table S2.** Device dimension according to gate length scaling.
